# Dielectrophoresis and ζ-potential as potential low-cost complements to patch clamp for measurement of cell electrophysiology

**DOI:** 10.1371/journal.pone.0356467

**Published:** 2026-08-20

**Authors:** Rashedul Hoque, Fatima H. Labeed, Srdjan Cirovic, Michael Pycraft Hughes

**Affiliations:** 1 Centre for Biomedical Engineering, University of Surrey, Guildford, United Kingdom; 2 Department of Biology, United Arab Emirates University, Al Ain, United Arab Emirates; 3 Department of Biomedical Engineering and Biotechnology, Khalifa University of Science and Technology, Abu Dhabi, United Arab Emirates; 4 Biotechnology Center, Khalifa University of Science and Technology, Abu Dhabi, United Arab Emirates; Complutense University of Madrid Faculty of Pharmacy: Universidad Complutense de Madrid Facultad de Farmacia, SPAIN

## Abstract

Cell electrophysiology is a useful measure of cell function, including ionic state and channel activity, and the way these are altered by pharmacological intervention. However, measurement of whole-cell electrical properties such as resting membrane potential *V*_*m*_ and membrane whole-cell capacitance and conductance is difficult, which has limited the more widespread adoption of electrophysiology in cell biology and drug discovery. The gold-standard method for acquiring this data is patch clamp, a method of measuring single cells that uses a micromanipulated micropipette in contact with the cell surface. Measurement is difficult, slow, and requires a highly skilled operator, whilst automated alternatives are expensive to run. Other methods such as flow cytometry are unable to provide absolute values of *V*_*m*_, or measure conductance and capacitance characteristics. Dielectrophoresis (DEP) and electrophoretic light scattering (ELS) have recently been shown to provide useful methods of directly measuring absolute *V*_*m*_ and other parameters, whilst examining many more cells (typically tens of thousands in under a minute). In this paper we use DEP and ELS to examine the electrophysiology of human bladder carcinoma cell line HTB-9 following treatment with ATP, glibenclamide and tetraethylammonium, and compare the results to published data obtained by patch clamp. Results indicate that DEP yields *V*_*m*_ and membrane conductance values that are highly comparable to data from patch clamp, at high speed and low cost, demonstrating the usefulness of techniques such as DEP and ELS for electrophysiological study.

## 1. Introduction

Cell electrophysiology describes the role of electrical phenomena in cell function [1]. As well as providing the physical basis for the function of excitable cells such as muscle and nerve, electrophysiological phenomena regulate many cellular functions in non-excitable cells through the use of ion channels which partition ions between the cytoplasm and extracellular space. Within the cytosol, these ions then regulate protein and organelle function. The quantity of ions passing through ion channels in the membrane can be measured as channel conductance; the diffusion potentials arising from unequal concentrations across the membrane gives rise to an electrical (membrane) potential *V*_*m*_, which then acts across the insulating component of the membrane as a capacitor. A key role of the study of cell electrophysiology study is to understand the effect of pharmaceutical interventions such as channel blockers on cell physiology. As well as playing a vital role in cell function, changes in cell electrophysiology are also associated with cell dysfunction. For example, cancer cells differ from their healthy counterparts in membrane potential [2] and membrane capacitance [3]; it has been shown that alteration of the cell’s electrical phenotype may play a role in cancer metastasis [4] and may offer a potential route to reverting to a pre-cancerous phenotype [5].

The gold standard method of measuring cellular properties such as the electrical potential, resistance and capacitance of the cell membrane is whole-cell patch clamp [6–8], wherein a current is measured through a saline-filled micropipette in contact with the cell cytoplasm via a small puncture in the membrane. Achieving this contact is a difficult and complex task; it requires identifying cells that can both provide a suitable seal and remain functional for the duration of the experiment, followed by careful micromanipulation of a micropipette tip to the surface of a cell a few microns across. Highly skilled operators are few and far between, and a skilled patcher can rarely measure more than a few cells per day. This in turn lends itself to operator bias, since only a small number of cells can be selected for patching, and that anecdotally these are typically the larger cells. Conversely, automated patch clamp systems [9] use microfluidic channels, where a reduced pressure and accompanying fluid flow draws single cells to a microscopic hole in the chip floor. This hole is then used to perform the patch. This allows tens of cells (typically 20–30) to be studied in parallel, and lack operator bias since no operator is involved in cell selection; however, these systems are often very expensive, with high initial purchase costs and expensive consumables.

Despite the importance of cell electrophysiology in areas such as cancer biology, the complexity and low throughput of single-cell analysis by patch clamp means that the field remains difficult to study for most laboratories. There is therefore a need to find alternative methods of electrophysiology assessment using low-cost, high-throughput, and ideally off-the-shelf instruments. Two approaches which have shown to offer potential as alternatives to patch clamp are dielectrophoresis (DEP), which measures the passive electrical properties of cells [10], and electrophoretic light scattering (ELS) which measures the ζ-potential, the electrical potential a short distance (ca. 1nm) outside the cell’s outer surface [11]. Both measure cell movement in electric fields to evaluate different electrical properties; both typically study tens of thousands of cells in under a minute, using disposable chips or cuvettes and requiring no operator skill beyond loading the consumables, making them much more widely amenable for cell electrophysiology than patch clamp. DEP uses non-uniform AC fields across a wide frequency range to determine a spectrum of mean cell movement. This “DEP spectrum” is then analysed using a theoretical model [12–14], with the best-fit model providing an estimated parameter set typically yielding the effective capacitance *C*_*eff*_ and conductance *G*_*eff*_ per unit area of the cell membrane, and the cytoplasm conductivity *σ*_*cyto*_. It has also been shown that analysis of DEP-derived *σ*_*cyto*_ as a function of medium conductivity *σ*_*med*_ yields a value of *V*_*m*_ that is highly comparable to patch clamp in cells ranging from blood to cancer [15], to yeast cells with different biofilm-forming capacity [16], to the transformation of primary chondrocytes into fibroblasts in culture [17]. Conversely, ELS has been used very infrequently in cell biology. It measures the extracellular surface potential at the cell’s hydrodynamic plane of shear, where water molecules and ions are no longer hydrostatically bound to the cell surface (typically around 1nm from the surface itself). This voltage, known as the ζ-potential, is widely used in solution chemistry to assess colloidal stability [18]. ELS measures this by observing the velocity of suspensoids in response to an imposed uniform DC field, by measuring the light scattered by the moving particles [11]. The standard model of ζ-potential is that it is generated purely by surface chemistry [18], but substantial evidence over decades [19] suggests it is altered by *V*_*m*_, most likely by capacitive coupling across the cell membrane and associated electrical double layers [20–23]. The movement of ζ-potential and *V*_*m*_ can be tracked in real time, for example in depolarised cardiomyocytes [24] and red blood cells suspended in low ionic strength media [25]. This effect allows cells to alter their interaction with their environment, including alteration of extracellular ion concentration [25], antibody-surface binding [26] and cell-surface adhesion [16]. Wider analysis of published data suggests potential roles from developmental biology to bacterial infectivity [19], but little work has been performed on ζ-potential and cancer with only one cancer cell type (MCF-7 breast cancer) having been reported [27] which showed ζ-potential depolarization compared to normal.

In this paper, we present a methodological study of the application of DEP (and to a lesser extent, ζ-potential) as an alternative to patch clamp. We selected the compounds to reflect those used in published studies of the same compounds on the same cell lines. The study of Monen [28], whilst not the only paper used as a reference, is a key comparative text; their work featured HTP-9 cells treated with Tetraethylammonium (TEA), adenosine triphosphate (ATP) and glibenclamide. TEA is generally considered an inhibitor of voltage-gated and large conductance Ca2 ^+^ -activated K^+^ channels, while glibenclamide and ATP inhibit ATP-dependent K^+^ channels. These treatments were selected by Monen et al [28] as part of a wider study of K^+^ currents in the bladder, which aligns with our work on bladder cancer electrophysiology [29,30]. Our results suggest that DEP yields values of *V*_*m*_ and membrane conductance which are highly comparable to data from patch clamp, but in a rapid and easy-to-use format that is readily amenable to the lab benchtop. This points towards the potential for widespread applicability of alternative methods to allow routine benchtop cell electrophysiology.

## 2. Materials and methods

### 2.1. HTB-9 cell culture and drug treatment

The HTB-9 bladder carcinoma cell line, also known as the 5637 cell line, was isolated from the urinary bladder of a 68-year-old male patient with grade II carcinoma in 1977 [31]. HTB-9 cells exhibit a high proliferation rate [32], capable of forming tumours [33] and have undergone extensive genomic characterization [34,35]. HTB-9 cells were obtained from ATCC, and no new human subjects or animal experiments were conducted. All cell culture supplements and chemicals were purchased from Sigma-Aldrich unless stated otherwise. Once thawed, HTB-9 cells were cultured in Roswell Park Memorial Institute RPMI-1640, modified with 25mM HEPES, and supplemented with 10% heat inactivated foetal bovine serum, 2mM L-glutamine and 1% penicillin-streptomycin incubated at 37°C, 5% CO_2_. The medium was replaced every 48−72 hrs and cells were passaged using trypsin-EDTA when reaching 70% confluency. HTB-9 cells were seeded 2.0 x 10^4^ cells/cm2 in T75 flasks. Once confluent, the harvested HTB-9 cells were centrifugated at 125 G, resuspended in fresh culture medium and equally split into four vials, corresponding to three treatment groups plus control.

### 2.2. MTT drug cytotoxicity assay

Concentrations of treatments were selected to allow comparison to published patch clamp studies [28,36], which were confirmed using MTT to determine the IC50 for each treatment. The MTT assay kit (Abcam) was used to evaluate the toxicity of K^+^ blockers TEA and glibenclamide (Fisher Scientific), as well as ATP nucleotide solution (Fisher Scientific). For each drug treatment, HTB-9 cells were seeded on a 96-well plate at a density of 2.0 X 10^4^ cells/well. TEA was added to each well to achieve a range of final concentrations (10^−1^ mM – 10^2^ mM), alongside untreated controls; the same was undertaken for glibenclamide and ATP (both 10^−2^ µM – 10^3^ µM). Cells were then incubated at 37°C for 24 hrs. After the incubation period, the treatment media was carefully aspirated and replaced with 50 µL of serum-free media and an equal volume of MTT reagent to each well. Additional background controls were set-up with the same volume of serum-free media and MTT reagent, with no cells. Cells were incubated at 37°C for 3 hrs. Viable cells with active metabolism were expected to convert MTT into formazan. After incubation, 150 µL of dimethyl sulfoxide (DMSO) was added to each well to dissolve formazan crystals, then covered and gently shaken at room temperature for 15 minutes. Absorbance was read at 590 nm via the SpectraMax iD3 (Molecular Devices) spectrophotometer. To determine the results of the MTT assay, the culture medium background control was subtracted from the absorbance reading of each sample to provide the corrected absorbance. Cell viability was then determined by the following equation:


$$\begin{array}{c} \%\;cytotoxicity\;=\;\frac{\left(\text{1}\text{0}\text{0}\;x\;(Control\;-\;Sample\right)}{Control} \end{array}$$ (1)

Cells without drug treatment were used as a control for cellular viability. Once dose-response curves were obtained, IC_50_ was determined for each drug treatment group. Dose–response curves were analysed using nonlinear regression in GraphPad Prism (GraphPad Prism 9 Software, San Diego, USA) using a four-parameter variable-slope model (“log(inhibitor) vs. response”). This performs a 4-way best-fit optimisation of the Hill model, using the data to find best-fit values for the maximum and minimum values, the Hill slope and the IC_50_, so that explicit predeterminations of the maximum and minimum values are not required. The IC_50_ was fitted with an R^2^ value of 0.99, giving a high degree of confidence in the estimated values. Further details can be found in the Supplementary Information. Once the optimum concentration was selected, cells were incubated with the three treatments for 2 hours prior to electrophysiological analysis.

### 2.3. DEP and ζ-potential experiments

Cells were analysed in a bulk isosmotic medium containing 248 mM sucrose, 16.7 mM dextrose, 250 μM MgCl_2_ and 100μM CaCl_2_. Osmolarity was measured (Osmomat 3000, Gonotec, US) at between 290–299 mOsm. Conductivity was adjusted to 43, 80 or 103 mS/m by adding phosphate buffered saline, verified using a Jenway 470 conductivity meter (Bibby Scientific). Cells were centrifugated at 125 G, and resuspended in fresh DEP medium, at a final cell concentration of 1 x 10^6^ cells mL^-1^ (± 15%). Previous studies have shown that there is no observable effect on the cell electrical properties due to detachment treatment [37].

Cells were analysed by DEP using a 3DEP cytometer (DEPtech, UK) [38]. Samples were analysed at frequency ranges between 10 kHz and 45 MHz at 10V peak-to-peak for 30 seconds. A minimum of three technical repeats were undertaken and the resultant DEP spectra was exported to Microsoft Excel for processing. The Malvern Panalytical Zetasizer Nano ZS90 (Malvern, UK) was used to measure the ζ-potential of HTB-9 cells. 800 µL of each treatment group sample, suspended in DEP medium, was injected into a disposable cuvette. A total of 4 biological repeats were undertaken for all study groups.

### 2.4. DEP cell characterisation

The HTB-9 cell population was counted using a haemocytometer and the average cell radius *r* of 100 cells was measured using Image J software (NIH, Maryland, US). For analysis, the DEP spectra of the technical repeats were averaged and a best fit (maximal R^2^) determined using a single-shell model [12–14] in Matlab (The Mathworks, USA). This yielded best-fit values effective membrane capacitance and conductance per unit area (*C_eff_* and *G_eff_* respectively), cytoplasm conductivity *σ_Cyto_*. Whole-cell capacitance (*C_w_*) was determined using Equation 2:


$$\begin{array}{c} C_{w}=\;{\text{4}\pi r^{\text{2}}\;C}_{eff} \end{array}$$ (2)

This has previously been shown to produce values comparable to whole-cell capacitance patch clamp data [39]. We also used *σ*_*cyto*_ to estimate *V*_*m*_ [15] using the equation:


$$\begin{array}{c} V_{m}=\;-\frac{RT}{F}\left|\text{ln}(A)\right|+\Psi_{ext}, \end{array}$$ (3)

Where *A* is the measured change in *σ*_*Cyto*_ for a given change in medium conductivity *σ*_*Cyto*_; *Ψ*_*ext*_ is the external surface potential, determined to be −12 mV in prior studies [15]; and *R*, *T* and *F* have represented their usual thermodynamic meanings.

### 2.5. Statistical analysis

Statistical analysis was performed using GraphPad Prism 9 software. Data are presented as mean ± SEM. Electrophysiological parameters (*C*_*eff*_*, G*_*eff*_ and *σ*_*Cyto*_) were compared between groups, as well as ζ-potential and modelled *V*_*m*_ values. Linear regression models were used to determine trends between continuous data sets. Tukey’s Honest Significant Difference (HSD) post hoc test to analyse pairwise differences. All variables were tested for normality. A t-test was performed to determine statistical significance between a specific treatment group and the untreated control group, if data were normally distributed. If one or both datasets were not normally distributed, a Mann-Whitney U-test was performed. Statistical significance was observed when the P-value was ≤ 0.05.

## 3. Results

**Fig 1:**
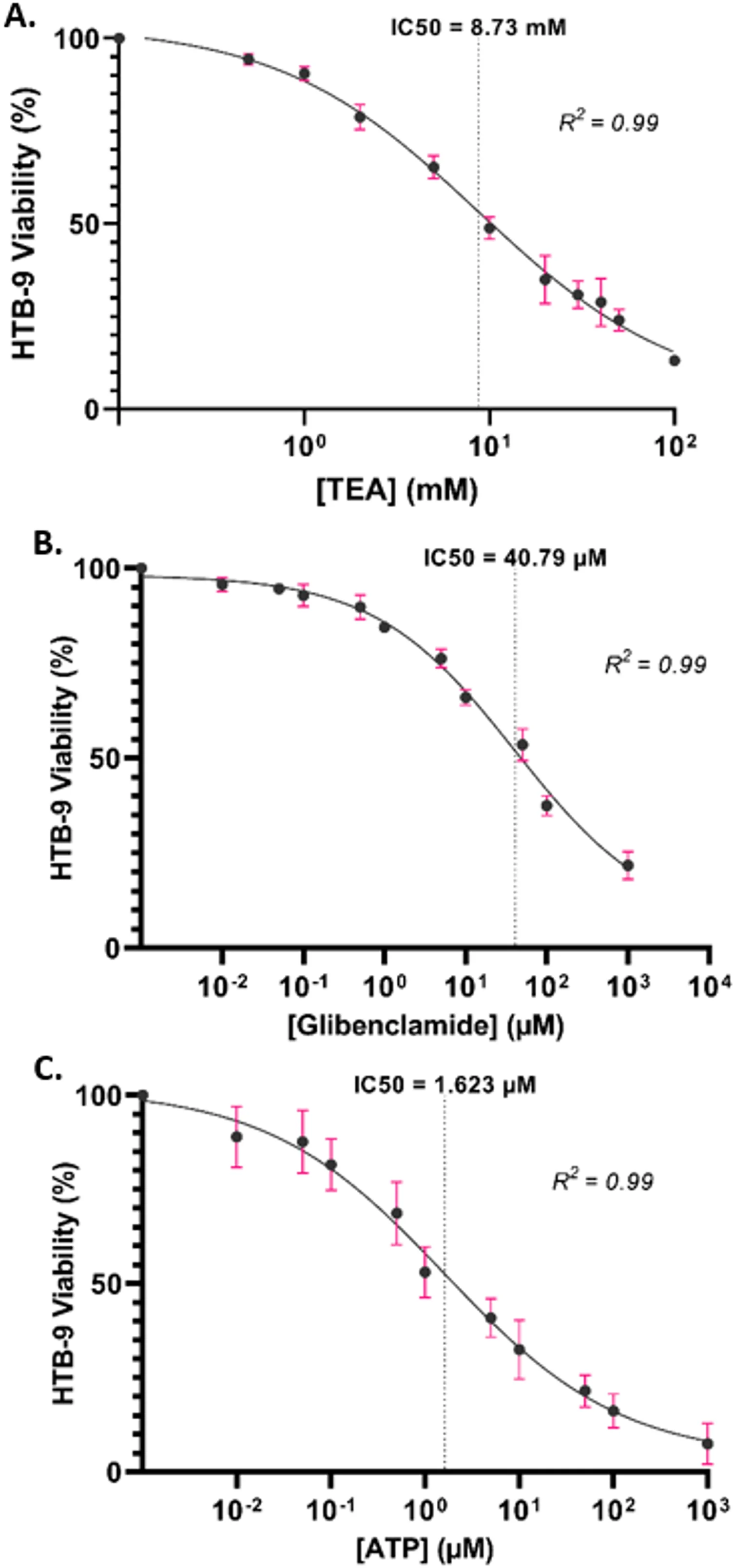
Incubation of HTB-9 cells for 24 hours with TEA, glibenclamide, or ATP reduces their viability in a dose-dependent manner. Drug-dose response/ drug cytotoxicity curves from MTT assay undertaken on HTB-9 cells, treated with: A.) TEA (10^−1^ mM – 10^2^ mM); B.) glibenclamide (10^−2^ µM – 10^3^ µM); C.) exogenous ATP (10^−2^ µM – 10^3^ µM). Dose–response curves were analysed using nonlinear regression in GraphPad Prism (GraphPad Prism 9 Software, San Diego, USA) using a four-parameter variable-slope model (“log(inhibitor) vs. response”). IC_50_ values were calculated directly from the fitted curves generated by the software.

The drug-dose response curves HTB-9 cells treated with TEA, glibenclamide and ATP are shown in Fig 1. The addition of TEA decreased cell viability in a dose-dependent manner, with an IC_50_ of 8.73 mM. Based on the experimental data, a dose of 10mM TEA was chosen to treat HTB-9 cells prior to DEP and ELS. This was the same concentration of TEA used to determine the electrophysiological changes in chondrocytes using the 3DEP system [13]. Furthermore, Monen et al. [28] demonstrated via patch-clamp analysis that a 10mM TEA treatment of HTB-9 cells reduced the open probabilities of calcium-activated potassium ion channels (KCa) to zero or near zero. The viability of HTB-9 cells was reduced with increasing concentrations of glibenclamide. Our measured IC_50_ for glibenclamide of 40.8 µM was comparable to that determined by Monen et al. [28] (46 µM), who employed a 50 µM dose of glibenclamide for subsequent investigations with the HTB-9 cell line. Thus, we selected 50 µM as the treatment dose for this study. Finally, cell viability declined following exogenous ATP treatment in a dose-dependent manner, with a recorded IC_50_ of 1.62 µM. Whilst Monen et al. [28] reported that increasing the concentration of exogenous ATP from 1 µM to 2mM abolished K^+^ channel activity, no dose-response curve or IC_50_ value was reported. A 10 µM concentration of ATP was chosen as an appropriate dose here.

HTB-9 cells are characterized by their ability to form adherent monolayers and showed an epithelial-like morphology, closely resembling the original tumour tissue [40]. Radii of around 100 cells were measured per treatment group; the mean radius of untreated cells was 8.0 ± 0.3 μm, whilst those treated with TEA and ATP had radii of 7.8 ± 0.1 and those treated with glibenclamide had radii 8.3 ± 0.1. There was no significant difference between untreated and any of the treated cells, and medium conductivity was not observed to influence cell size.

**Fig 2:**
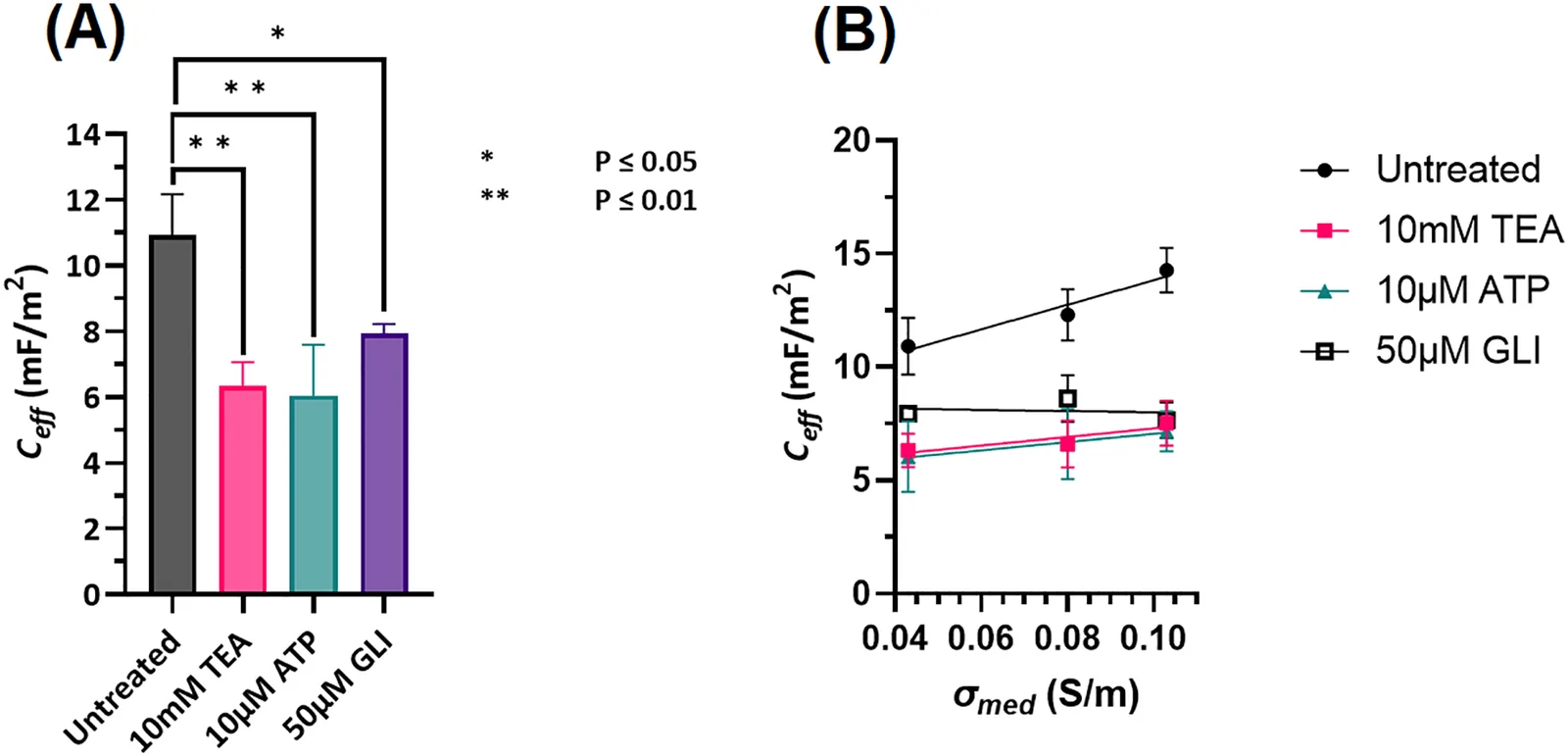
Treatment of HTB-9 cells with TEA, glibenclamide, or ATP significantly decreases the effective membrane capacitance, as determined by DEP. Effective membrane capacitance (*C_eff_*) of HTB-9 cells at (A) 43 mS/m and (B) as a function of medium conductivity, either untreated or treated with 10mM TEA, 10 µM ATP or 50 µM glibenclamide. T-tests were undertaken to determine statistically significant differences between untreated HTB-9 cells and each treatment group. Data shown as mean ± SEM, n = 4 biological replicates, each with >3 technical replicates.

The DEP and ζ-potential data showed observable, statistically significant changes in the passive electrophysiological parameters of HTB-9 cells in the three treatment groups (TEA, glibenclamide and ATP) compared to untreated controls. The *C_eff_* of the control and three treatments at 43 mS/m is shown in Fig 2. The average *C_eff_* of the untreated HTB-9 cells was 10.9 ± 1.3 mF/m^2^ and was significantly reduced with 10 mM TEA (6.33 ± 0.74 mF/m^2^, P = 0.002), 50 µM glibenclamide (7.94 ± 0.28 mF/m^2^, P = 0.016) and 10 µM exogenous ATP (6.04 ± 1.56 mF/m^2^, P = 0.003). Tukey’s HSD identified a significant difference in *C_eff_* between the untreated group and TEA (P < 0.001); glibenclamide (P < 0.01) and ATP (P < 0.001). There was no significant difference in *C_eff_* identified between the three treatment groups. Similar trends were observed in higher conductivity media; the relationships remained broadly the same, though the mean capacitance was observed to increase with conductivity from 10.9 mF/m^2^ at 43 mS/m to 12.3 mF/m^2^ at 80 mS/m and 14.3 mF/m^2^ at 103 mS/m.

When the capacitance values were converted to whole-cell values (Equation 2), the mean *C*_*w*_ of untreated HTB-9 cells was calculated at 8.7 ± 1.2 pF; after treatment with TEA this dropped to 4.7 ± 0.6 pF, treatment with glibenclamide was 6.8 ± 0.4 pF, and after treatment with ATP was 4.5 ± 1.2 pF. The treatment group were significantly lower than the untreated cells (*P < 0.05* for all). Tukey’s HSD identified a significant difference in *C*_*w*_ between the untreated group and TEA (*P* = 0.0033) and ATP (*P* = 0.0026). Interestingly, Tukey’s HSD identified no significant difference between the *C*_*w*_ of untreated HTB-9 cells and those treated with 50 µM glibenclamide (P = 0.043) but showed a significance comparing the *C*_*w*_ of glibenclamide to 10 mM TEA and 10 µM groups (*P <* 0.025 for both comparisons). This was likely due the *C*_*w*_ of the glibenclamide-treated group being still lower than the untreated group but noticeably more elevated than the other treatment groups.

**Fig 3:**
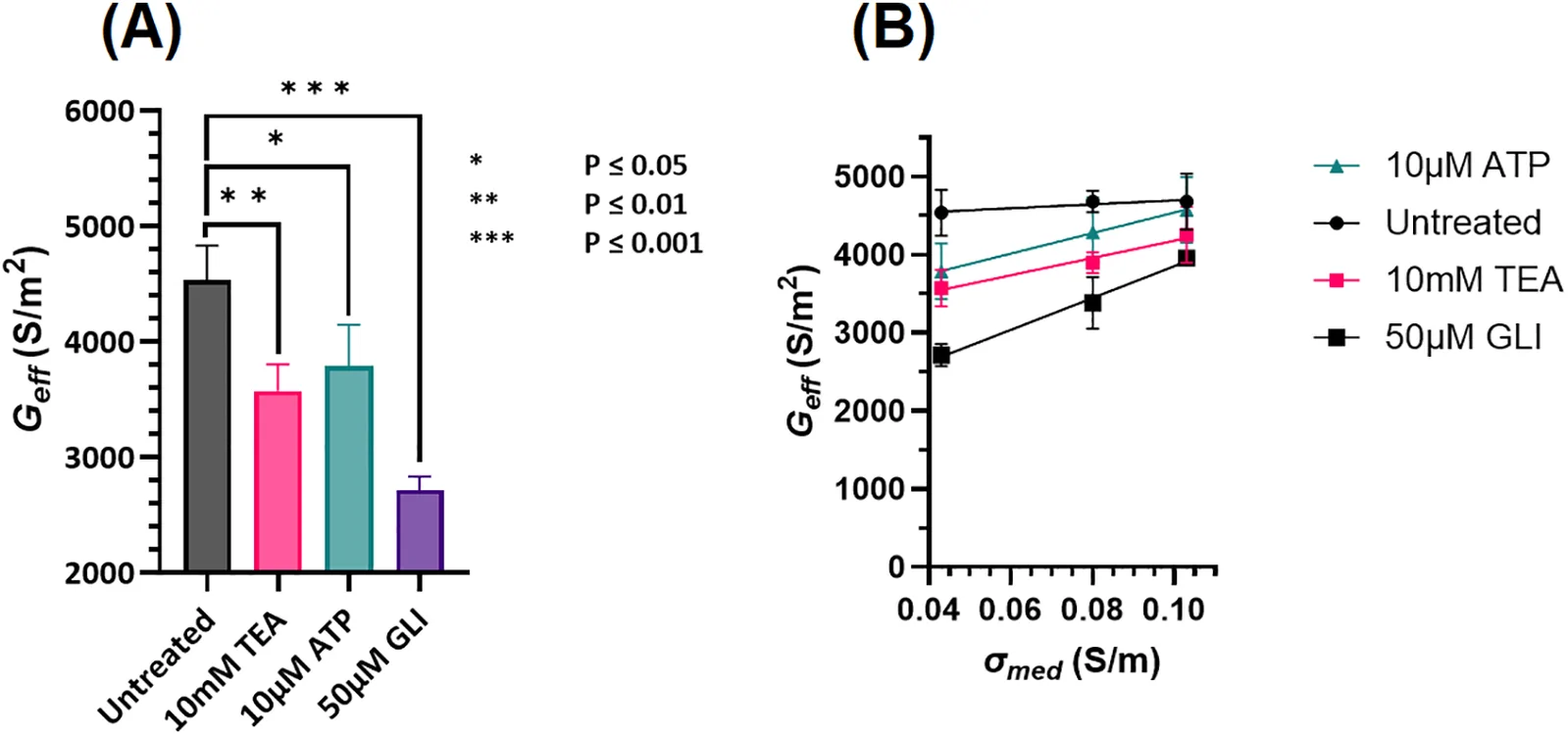
Treatment of HTB-9 cells with TEA, glibenclamide, or ATP significantly decreases effective membrane conductance, as determined by DEP. Effective membrane conductance (*G_eff_*) of HTB-9 cells at (A) 43 mS/m, and (B) as a function of medium conductivity, either untreated or treated with 10mM TEA, 10 µM ATP or 50 µM glibenclamide. Pairwise t-tests with Welch’s correction were undertaken to determine statistically significant differences between untreated HTB-9 cells and each treatment group. Data shown as mean ± SEM, n = 4 biological replicates, each with >3 technical replicates.

The *G*_*eff*_ values of all study groups in 43 mS/m medium are shown in Fig 3a. *G*_*eff*_ of untreated HTB-9 cells in 43 mS/m solution was 4.53 ± 0.29 kS/m^2^, which significantly decreased when treated with 10mM TEA (3.57 ± 0.23 kS/m^2^, *P = 0.003*), and 10 µM ATP (3.79 ± 0.36 kS/m^2^, *P = 0.019*) and 50 µM glibenclamide (2.71 ± 0.12 kS/m^2^, *P = 0.0004*). One-way ANOVA showed a highly significant difference between study groups (F (3, 12) = 31.6, P < 0.0001). Tukey’s HSD identified a significant difference between the untreated group and TEA (*P < 0.01*), ATP (*P < 0.01*) and glibenclamide (*P < 0.001*). Pairwise comparisons were also observed between glibenclamide and the other treatment groups (*P < 0.01* for both comparisons). When the trend was measured with increasing medium conductivity, we found that all four trended towards a common value (Fig 3b), with significant difference not being observed at higher conductivity.

**Fig 4:**
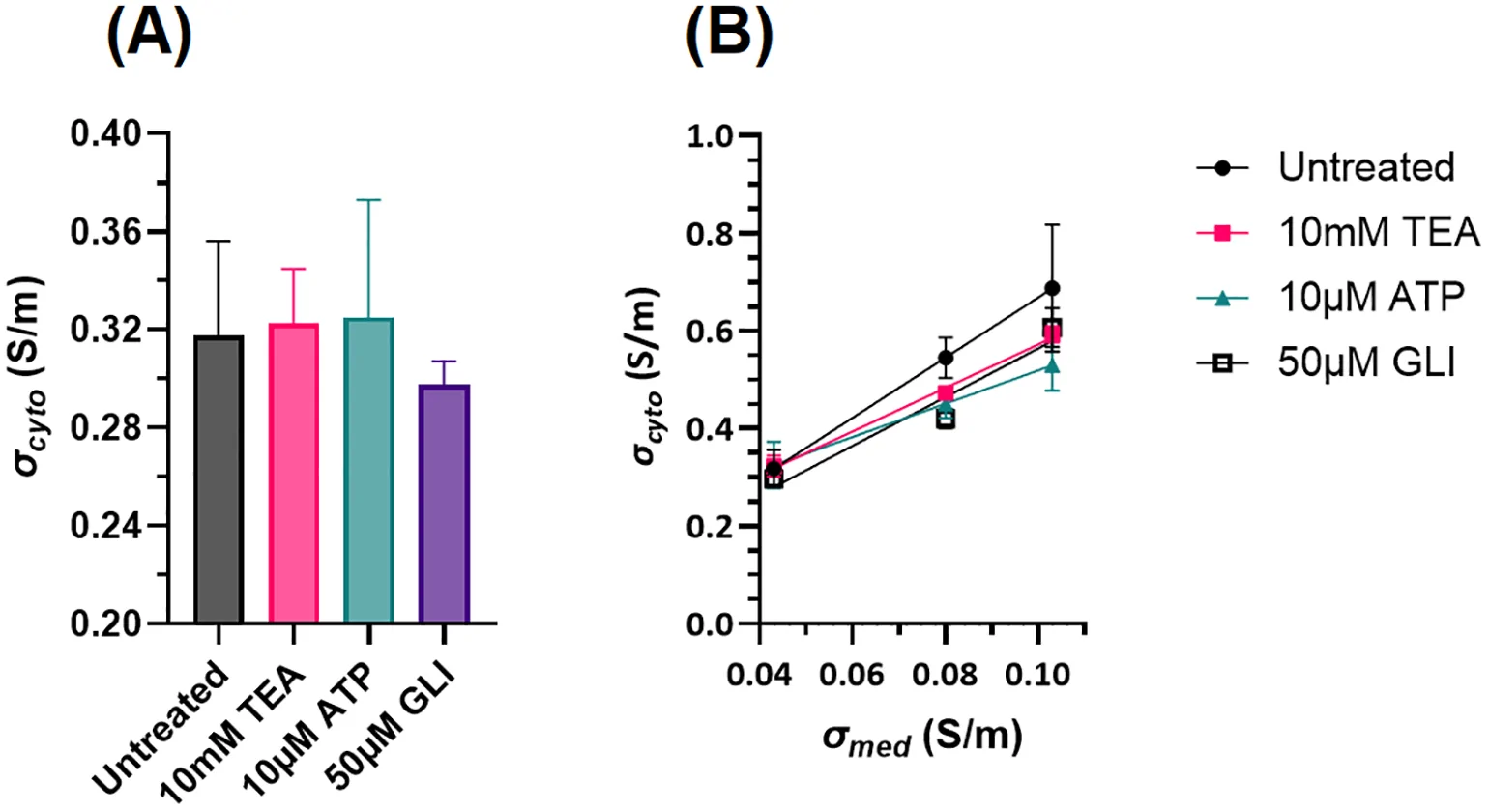
Cytoplasmic conductivity of HTB-9 cells varies linearly with medium conductivity. The cytoplasmic conductivity (*σ_Cyto_*) of HTB-9 bladder carcinoma cells, either untreated or treated with 10mM TEA, 10 µM ATP or 50 µM glibenclamide. Investigations were undertaken at (A) 43 mS/m and (B) as a function of medium conductivity. Pairwise t-tests with Welch’s correction were undertaken to determine statistically significant differences between untreated HTB-9 cells and each treatment group. Data shown as mean ± SEM, n = 4 biological replicates, each with >3 technical replicates.

There was no significant difference in the *σ*_*Cyto*_ values (Fig 4a) between the untreated HTB-9 cells (0.32 ± 0.04 S/m) and cells treated with 10 mM TEA (0.32 ± 0.02 S/m), 10 µM ATP (0.33 ± 0.05 S/m) and 50 µM glibenclamide (0.30 ± 0.01 S/m, P > 0.05 for all groups) at the standard medium conductivity of 43 mS/m. However, notable changes were detected at a conductivity of 80 mS/m; at this higher medium conductivity, the *σ*_*Cyto*_ of untreated HTB-9 cells was 0.55 ± 0.04 S/m, which significantly reduced when treated with TEA (0.47 ± 0.01 S/m, *P = 0.036*), exogenous ATP (0.45 ± 0.03 S/m, P = 0.012) and glibenclamide (0.42 ± 0.02 S/m, P = 0.004). As can be seen in Fig 4b, *σ*_*cyto*_ of all cells exhibits a divergent linear gradient with increasing *σ*_*med*_.

**Fig 5:**
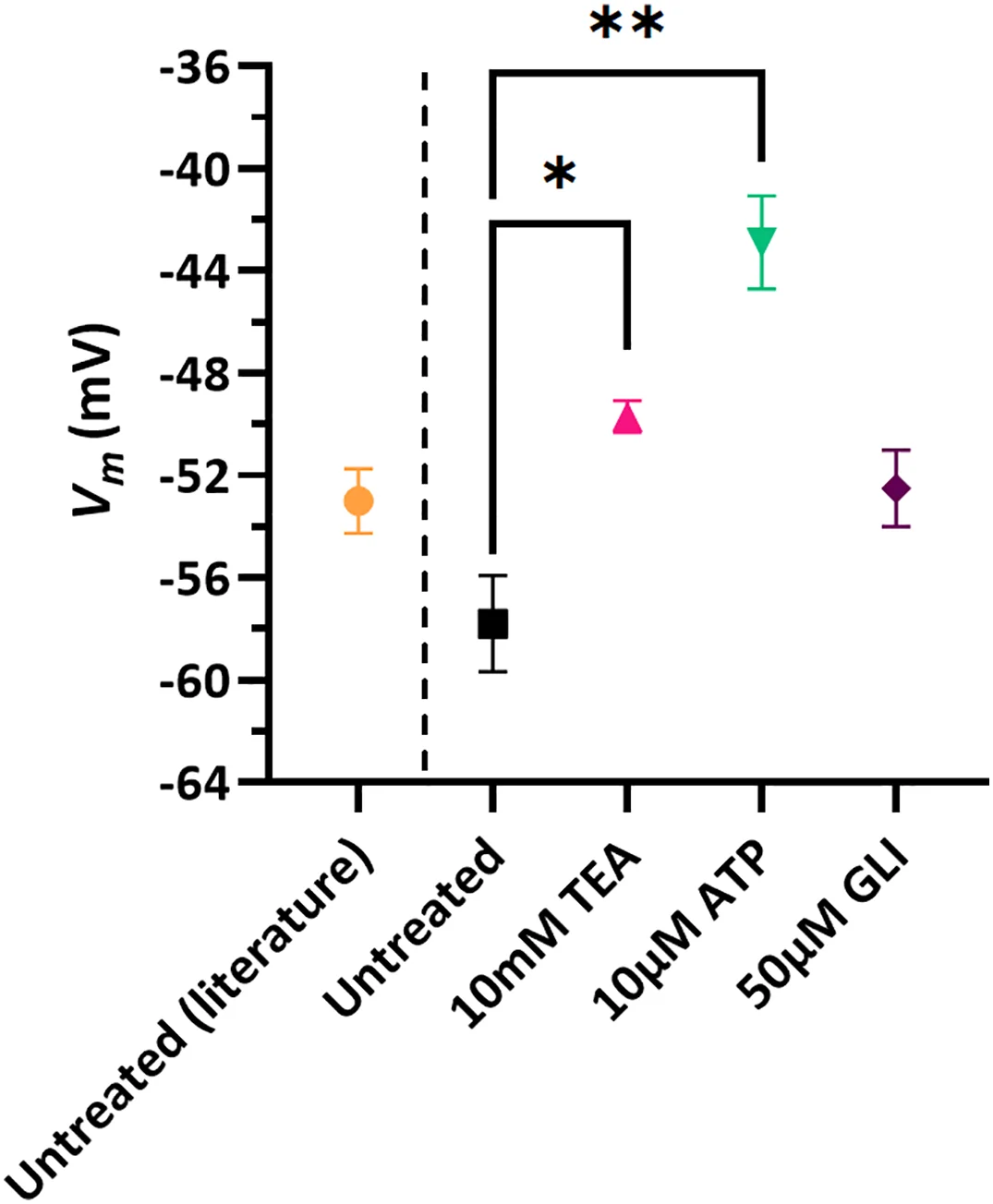
TEA and ATP depolarize membrane potential of HTB-9 cells, but glibenclamide does not. DEP-derived *V_m_* values of HTB-9 bladder carcinoma cells, either untreated or treated with 10mM TEA, 10 µM ATP or 50 µM glibenclamide. The measured value of *V_m_* of untreated HTB-9 cells is plotted for comparison, based on the published findings of Monen et al. [33]. Pairwise t-tests with Welch’s correction to determine significance. Data shown as mean ± SEM, n = 4 biological replicates, each with >3 technical replicates.

The slopes from the linear regression models shown in Fig 4b were used in Equation 3 to estimate *V*_*m*_ of each study group. These are shown in Fig 5, together with patch clamp measured data of untreated HTB-9 cells from literature for comparison [28]. The *V*_*m*_ of untreated HTB-9 cells calculated from the model was −57.8 ± 3.8 mV. In comparison to untreated controls, there was a significant depolarization in the modelled *V*_*m*_ of HTB-9 cells treated with 10mM TEA (−49.7 ± 1.2 mV, P = 0.018) and 10 µM ATP (−42.9 ± 3.6 mV, P = 0.0013). A non-significant depolarisation was observed following glibenclamide treatment (−52.5 ± 3.0 mV). Tukey’s HSD did not identify any significant pairwise comparisons (P > 0.05 for all). However, when comparing the measured *V*_*m*_ from the literature to the ATP treated group, a significant difference was identified (P = 0.031). The data suggests that treating HTB-9 cells with TEA or ATP causes membrane depolarization, whilst glibenclamide does not.

**Fig 6:**
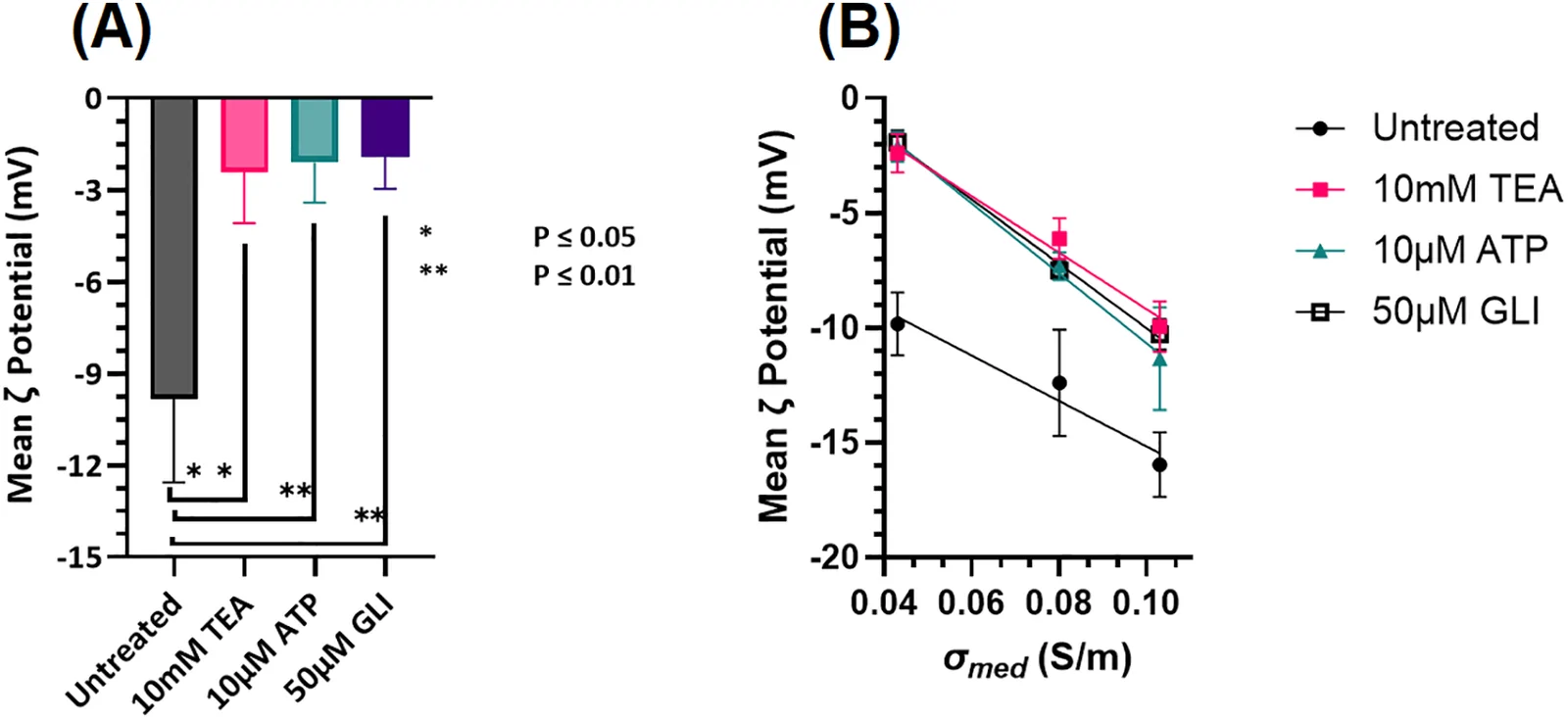
The ζ-potential of HTB-9 cells differs after treatment and polarizes linearly with medium conductivity. The ζ-potential of HTB-9 bladder carcinoma cells, either untreated or following treatment with 10mM TEA, 10 µM ATP or 50 µM glibenclamide at (A) 43 mS/m and (B) as a function of medium conductivity. Data shown as mean ± SEM, n = 4 biological replicates, each with >3 technical replicates. Pairwise t-tests with Welch’s correction were used to determine significance.

The ζ-potential of untreated HTB-9 cells at 43 mS/m was measured using ELS at −9.8 ± 2.7 mV (Fig 6a). The ζ-potential significantly depolarised when cells were treated with 10mM TEA (−2.4 ± 1.7 mV), 10 µM ATP (−2.1 ± 1.3 mV) and 50 µM glibenclamide (−1.9 ± 1.1 mV, P < 0.01 for all treatment groups). Tukey’s HSD identified a significant difference between the ζ-potential of untreated HTB-9 cells and all treatment groups (P < 0.001 for all). There was no significant difference in the pairwise comparisons *between* treatment groups, with all three treatments producing similar effects. Increasing the medium conductivity saw the ζ-potential of all four cell conditions becoming more polarised (Fig 6b), but with the relationship between them remaining similar with significant differences between untreated cells and all treatments at 80 mS/m, and all except TEA at 103 mS/m.

## 4. Discussion

Given that patch clamp remains the gold standard of cell electrophysiology, it is rational to begin by comparing the results taken by DEP and ELS with those acquired using patch clamp under similar conditions. For example, Monen et al. [28] reported mean resting membrane potential of −53 ± 4.9 mV based on recordings from 15 cells. This compares with our estimated value of −56.1 ± 10.5 mV; the increased range of variation is likely due to the substantially larger numbers of cells measured (typically around 60,000 across multiple technical repeats, compared to 15 cells by Monen et al [28]). The measured whole-cell capacitance of untreated THB-9 cells by DEP was 8.7 ± 1.2 pF for untreated cells, which is lower than the reported value by patch clamp of 20 ± 1.1 pF [28]. This observation of lower DEP-generated *C*_*w*_ is consistent with previous reports comparing DEP-acquired measurements with those from patch clamp [38]. Specifically, where cells are homogeneous in size (e.g., red blood cells) DEP and patch clamp yield very similar values of *C*_*w*_. Conversely, where cell size is more heterogeneous, DEP reports lower *C*_*w*_ than patch clamp. This has been attributed to operator bias in cell selection, a widely reported issue with patch clamp (e.g., [41]), for example favouring larger cells over smaller ones. DEP measures all cells across the entire size range, leading to a much more representative value. An average difference in radius of around 25% between the Monen-patched cells and the complete cell population described here would account for the observed differences.

When we considered cell membrane capacitance per unit area *C*_*eff*_ (10.9 ± 1.3 mF/m^2^) we found that it was similar to other reported cancer cell lines, including breast cancer [42,43], colon cancer [44], melanoma [44], leukaemia [45,46] and osteosarcoma [47], all of which range from 8.2 to 13.6 mF/m^2^. This suggests that the values of *C*_*eff*_, and by extension *C*_*w*_, are valid across the whole cell population. We can take a similar approach to that used for capacitance in Equation (2) to determine the whole-cell resistance *R*_*w*_, by multiplying the effective membrane resistance (equal to 1/*G*_*eff*_) by the surface area of the cell. When we compared our treatment groups to control, we found that all three exhibited a drop of *C*_*eff*_ from control (around 10–15 mF/m^2^) to between 6–9 mF/m^2^. It is known that the lipid bilayers cells have a value of around 8 mF/m^2^ when flat [48]. The DEP model assumes a flat surface, but a cell with folds and blebs will have a larger actual area that that predicted from a calculation for a geometric sphere of the same radius. Hence the ratio of actual to effective capacitance has been described by Gascoyne et al [49] as the folding factor *φ*, where the net effect of increased membrane folding is an increase in *effective* membrane capacitance over the *actual* capacitance of a flat membrane of the same composition. Our results suggest that the use of the three interventions yielded smoother membranes, potentially due to alterations in cytoplasm content altering water content and small variations in cell swelling. Another interesting observation is that only control cells exhibited a change in *C*_*eff*_ as a function of medium conductivity, a phenomenon which has also been observed in red blood cells [25], which may be related to the higher value of membrane potential in these cells altering the electrical double layer composition; the electrical double layer capacitance Is related to the double layer thickness, which is in turn related to the cell surface charge [18].

As expected, the measured value of *R*_*w*_ (276 kΩ) is substantially lower than that measured by patch clamp, where the reported *R*_*w*_ was 404 ± 55 MΩ [28]. This is due to the resistance measured by patch clamp consisting only of the resistance through the membrane, whereas the value measured by DEP also includes conduction through the electrical double layer in parallel [14,18].

The medium conductivity, reflecting the ionic strength in the suspending medium was observed to impact all the electrical measurements here. It has been known for some time [10,50,51] that DEP response is affected by medium conductivity, with alterations in the frequency-dependent DEP response observed across various cell types including human colon cancer [52], melanoma [53], and breast cancer cells [53], when suspended in different conductivity media. Whilst the change in one of these parameters (*σ*_*Cyto*_) is the basis of Equation 3, changes were also observed in other parameters. Cell membrane capacitance was observed to increase across the three conductivities for untreated cells only; no increase was observed in the three treatment cases.

Conversely, a clear linear increase was observed in *G*_*eff*_ for all treatment cases, but not for control cells. The linear increase in *G*_*eff*_ tallies with observations in red blood cells, platelets, monocytes, neurons and cervical cancer cells [54,55] which suggested that double-layer conduction dominates the *G*_*eff*_ parameter, and that this is reduced by more polarised membrane potentials countering the surface charge through capacitive interactions causing induction of positive charge. This in turn aligns with the estimated values of *V*_*m*_ from the Results. We observed a significant reduction in *G*_*eff*_ following TEA and glibenclamide treatments. Whilst such a change could be associated with a change in cell size and commensurate change in surface area, no significant change was observed in radii. This being discounted, the remaining possibilities are that the change reflects the transmembrane conductance, or to reduced conduction in the electrical double layer.

We can consider this in light of data reported by Monen et al [28], who reported values of specific conductance changes for some of their treatments. For 50 μm glibenclamide, the specific conductance reduced by 58.5% (from 0.65 to 0.27 nS/pF) after treatment. For the nonspecific K^+^ channel blocker charybdotoxin, the actual conductance was not reported. However, analysis of the plots of transmembrane current vs *V*_*m*_ suggests that treatment resulted in a reduction of approximately x6 compared to control. When we compared these results to ours, we found that at 43 S/m, TEA reduced *G*_*eff*_ by 22%, whereas glibenclamide reduced it by 40%.

If the membrane conductance comprises two components, then we can attempt to separate the component associated with the membrane from that associated with the double layer. It is clear from Fig 3b that there is a component of *G*_*eff*_ that relates to the conductivity of the suspending medium, resulting in the linear increase between *G*_*eff*_ and *σ*_*med*_. We can account for this by considering the y-intercept of Fig 3b, which would suggest the value of conductance when the conductivity-related component is zero. In this instance, the reduction in *G*_*eff*_ compared to untreated cells due to TEA, ATP and glibenclamide are 31%, 27% and 59% respectively. This last value shows a high degree of similarity to the 58.5% observed by Monen et al. [28] and suggests that estimation of baseline *G*_*eff*_ for zero *σ*_*cyto*_ has potential for accurate determination of pharmacological effect on membrane conductance.

When cytoplasm conductivity was considered, we found that at 80 mS/m all treatment groups exhibited significantly reduced *σ*_*Cyto*_ compared to control, indicative of reduced ionic strength in cells, but the difference between controls and glibenclamide treated HTB-9 cells was the greatest (0.55 ± 0.04 S/m versus 0.42 ± 0.02 S/m, P = 0.004). From Equation (3), evaluation of *σ*_*cyto*_ is a necessary component for determining *V*_*m*_ by DEP. Whilst Monen et al. [28] reported baseline *V*_*m*_ for untreated cells, they did not use whole-cell patch clamping to determine *V*_*m*_ after pharmacological treatment, relying instead on measurement of ion currents. For example, as the primary ion in membrane potential maintenance, a reduction in intracellular K^+^ leads to membrane depolarization [1]. When we analysed DEP-derived *V*_*m*_, a statically significant 8.1 mV depolarisation (P = 0.0013) was observed after the application of TEA. Glibenclamide yielded a small (5.3 mV), non-significant change. This is in line with analysis of other cells; for example, myocytes treated with glibenclamide showed little or no change in *V*_*m*_ [56,57]. This is possibly due to its dual action as a blocker of both K^+^ and Cl^-^, with the depletion of both acting to cancel out contributions to *V*_*m*_ from the two components. The greatest reduction in *V*_*m*_ (23%) occurring in ATP-treated cells, in line with Monen et al’s [28] observation that extracellular ATP eliminated all channel activity.

Substantial changes were observed in ζ-potential, particularly at lower conductivities where all three treatment cases showed significant depolarisations compared to control. Since none of the interventions are expected to alter the surface chemistry of the cells, this suggests that the observed depolarisation may be driven by the coupling of *V*_*m*_ across the membrane. This phenomenon has been reported in a number of cells, including red blood cells [25], platelets [26], yeast cells [16], and cardiomyoblasts [24]. This effect in turn may be affected by *V*_*m*_ itself, or by the degree of capacitive coupling, or most likely a combination of the two, since treatment in all cases produced both *V*_*m*_ depolarisation and a reduction in *C*_*eff*_. Bondar et al. [58], reported that the ζ-potential of HeLa cells became significantly more negative after being subjected to heat shock, which was attributed to the redistribution of negatively charged phosphatidylserine, a marker for apoptosis. This suggests that the depolarisation of ζ-potential of HTB-9 after treatment with TEA, glibenclamide and ATP was not the result of cell death. Instead, the treatments likely influenced the homeostatic mechanisms of HTB-9 cells to maintain stability.

The increase in ζ with *σ*_*med*_ is perplexing, as conventional models of ζ-potential behaviour suggest that the parameter should depolarise with increasing medium ion concentration, since the electrical double layer decreases in size with increased bulk charge, but the ζ-potential is measured at a point typically 1–2 nm outside the cell surface, whose position does not move with conductivity. Here we see a linear *increase* in cell polarisation. This preliminary result requires future dedicated study, but also suggests that whilst ζ-potential may have further potential in electrophysiological analysis, it may be more suited to a binary, diagnostic role than to a measurement role.

## 5. Conclusion

Electrophysiology plays an important role in cellular function but has not received the attention is deserves due to difficulties and costs associated with direct cell measurement which have limited the widespread adoption of electrophysiology as a general tool for cell biology. This has in turn reduced the opportunities for developing a deeper understanding of the importance of electrophysiology in cell function. In this paper, we have demonstrated that DEP and ζ-potential can provide new insights to cell electrophysiology, and in the case of DEP, can replicate patch-clamp derived values of *V*_*m*_ of HTB-9 cells, whilst relative changes in membrane conductance after treatment with glibenclamide match the reported data to within 0.5%.

## Supporting information

S1 Data Contains the data used in this paper. (PDF)

S1 File Contains detailed information about the determination of IC50. (DOCX)
