# Supplementary material for: Dielectrophoresis and ζ-potential as potential low-cost complements to patch clamp for measurement of cell electrophysiology
Source: PLoS One. 2026 Aug 20;21(8):e0356467. doi: 10.1371/journal.pone.0356467 (PMC13492762; doi:10.1371/journal.pone.0356467)
Supplement: Supplementary file 1 [file pone.0356467.s001.pdf]

	MEDIUM CONDUCTIVITY (S/m)	Untreated				10mM TEA				10μM ATP				50μM GLI			
C_{eff} (mF/m^2)	0.043	12.4	10.37	9.49	11.38	6.96	5.57	6.96	5.82	7.34	6.7	6.32	3.79	7.842	7.589	8.222	8.095
	0.08	12.02	11.26	12.02	13.91	7.21	5.06	7.21	6.96	8.1	7.08	6.83	4.43	7.589	9.486	9.486	7.842
	0.103	15.18	13.91	13.03	14.93	7.84	8.73	7.08	6.45	8.35	7.08	6.96	6.2	7.716	7.842	8.475	6.577
G_{eff} (S/m^2)	0.043	4571.4	4857.1	4571.4	4142.9	3571.4	3571.4	3857.1	3285.7	3428.6	4285.7	3714.3	3710	2857.1	2714.3	2571.4	2714.3
	0.08	4571.4	4714.3	4857.1	4571.4	3714.3	4000	4000	3857.1	4000	4857.1	4428.6	3857.1	3000	3571.4	3571.4	3380.9
	0.103	4142.9	4857.1	4857.1	4857.1	4142.9	4285.7	4714.3	3857.1	4285.7	4857.1	5000	4142.9	3857.1	4000	4000	3714.3
Zeta (mV)	0.043	-13.608	-10.046	-7.885	-7.78	-0.019	-3.197	-3.8255	-2.6145	-1.586	-2.283	-3.8255	-0.7919	-0.64	-1.963	-1.8675	-3.204
	0.08	-12.97	-18.64	-7.862	-10.143	-5.6345	-5.696	-8.626	-4.52	-6.538	-6.045	-8.626	-8.0425	-6.88	-7.345	-7.135	-8.64
	0.103	-15.223	-18.02	-18.275	-12.31	-11.633	-9.266	-7.134	-11.765	-13.77	-7.088	-8.134	-16.4	-10.555	-9.847	-8.73975	-11.961
σ_{med} (S/m)	0.043	0.26	0.33	0.34	0.34	0.3	0.33	0.31	0.35	0.28	0.33	0.3	0.39	0.31	0.29	0.29	0.3
	0.08	0.6	0.55	0.53	0.5	0.46	0.49	0.47	0.47	0.44	0.42	0.45	0.49	0.42	0.4	0.44	0.4
	0.103	0.87	0.69	0.61	0.58	0.6	0.61	0.57	0.59	0.57	0.52	0.46	0.57	0.65	0.6	0.57	0.51
Radius (μm)	0.043	8.2365	7.8785	8.1125	7.689	7.5475	7.6235	7.8395	7.7015	7.7645	7.576	7.7325	7.692	8.231	8.172	8.242	8.449
	0.08	7.545	8.0455	8.2355	8.1275	7.8545	7.6705	7.8505	7.7745	7.813	7.854	7.8755	7.6875	8.284	8.026	8.222	8.394
	0.103	7.6135	8.2005	8.1795	8.1515	7.994	7.7265	7.902	7.6005	7.8325	7.8925	7.7975	7.703275	8.312	8.129	8.234	8.448
