# Supplementary material for: Dielectrophoresis and ζ-potential as potential low-cost complements to patch clamp for measurement of cell electrophysiology
Source: PLoS One. 2026 Aug 20;21(8):e0356467. doi: 10.1371/journal.pone.0356467 (PMC13492762; doi:10.1371/journal.pone.0356467)
Supplement: Supplementary file 2 [file pone.0356467.s002.docx]

**Determination of IC50 values.**

IC50 values were determined using the dose-response curves shown in Figure 1. Dose–response curves were analysed using nonlinear regression in GraphPad Prism (GraphPad Prism 9 Software, San Diego, USA) using a four-parameter variable-slope model (“log(inhibitor) vs. response”) to determine the IC_50_ for each drug treatment group.

In order to determine the IC50, a linear regression was performed to identify the best-fit parameter set between the measured data and the Hill equation:

$$Y=Bottom+\left( \frac{Top-Bottom}{1+{10}^{((LogIC50-X)\times HillSlope)}} \right)$$

Where *Y* is the dose response, *Top* and *Bottom* are the values of viability in the plateaux at the start and end of the curve, the *HillSlope* is the steepness of the curve, the *IC50* represents the 50% inhibitory concentration, and *X* is the dose used. All four parameters were freely estimated, without constraints. The IC50 was fitted with an R^2^ value of 0.99, giving a high degree of confidence in the estimated values.

The parameter sets determined by this model, together with 95% confidence intervals, can be seen in Supplementary Table 1. The estimates for ATP and TEA show relatively small confidence intervals, suggesting a high level of confidence in the IC50 parameter; for glibenclamide, the dose-response bure did not reach a saturating lower plateau, resulting in a wider confidence interval.

The aim of the IC_50_ study was not to determine definitive values of IC50 for these drugs with the HTB-9 cell line, but to validate the selection of dosage used in the study of Monen et al [1], which is replicated here to allow comparability of results. As an be seen in the table, the measured IC50 of ATP and TEA closely align with the concentrations used in the prior work, whilst the glibenclamide value is slightly lower than the values measured by Monen et al [1], it is sufficiently close to the value used to justify the use of the concentration in the paper.

|  | **Monen study** | **Monen IC50** | **IC50** | **Top (%)** | **Bottom (%)** | **Hill Slope** |
| --- | --- | --- | --- | --- | --- | --- |
| **ATP** | 1-2 µM | NA | 1.62 µM  (0.838 to 3.46) | 101.7 (93.83 to 114.2) | 3.41  (-12.8 to 12.7) | -0.463  (-0.640 to -0.314) |
| **TEA** | 10 mM | NA | 8.73 mM  (6.51 to 13.6) | 103.5  (98.5 to 110) | 2.98  (-14.8 to 12.7) | -0.804  (-1.02 to -0.608) |
| **Gli** | 50 µM | 46 1 µM | 40.8 µM  (24.2 to 97.1) | 98.3  (95.6 to 101) | 5.47  (-12.9 to 15.7) | -0.501  (-0.620 to -0.391) |

**Supplementary Table 1.**  The best-fit IC50, Top, Bottom and Hillslope parameters (plus 95% confidence intervals in parentheses) determined using linear regression in GraphPad Prism, together with the IC50 reported by Monen et al., and the concentrations used experimentally in that paper.

[1] Monen SH, Schmidt PH, Wondergem R. Membrane Potassium Channels and Human Bladder Tumor Cells. I. Electrical Properties. Journal of Membrane Biology 1998;161:247–56. <https://doi.org/10.1007/s002329900331>.
